# Supplementary material for: Mapping the evolution of fertility support policies in China: A content and instrumental analysis
Source: PLoS One. 2025 Oct 9;20(10):e0332137. doi: 10.1371/journal.pone.0332137 (PMC12510515; doi:10.1371/journal.pone.0332137)
Supplement: S1 Appendix — (ZIP) [file pone.0332137.s001.zip › S1 Appendix. 226 original policy documents/208-第十三届全国人民代表大会财政经济委员会关于国民经济和社会发展第十四个五年规划和2035年远景目标纲要草案的审查结果报告(FBM-CLI-1-353495).doc]

第十三届全国人民代表大会财政经济委员会关于国民经济和社会发展第十四个五年规划和2035年远景目标纲要草案的审查结果报告

**发布部门：** [全国人大财政经济委员会](https://www.pkulaw.com/law/chl?Aggs.IssueDepartment=105)

**发布日期：**2021.03.09

**实施日期：**2021.03.09

**时效性：** [现行有效](https://www.pkulaw.com/law/chl?Aggs.TimelinessDic=01)

**效力级别：** [工作文件](https://www.pkulaw.com/law/chl?Aggs.EffectivenessDic=XA0105)

**法规类别：** [人大议事](https://www.pkulaw.com/law/chl?Aggs.Category=00106)

**专题分类：** [依法治国](https://www.pkulaw.com/law/chl?Aggs.SpecialType=019)

第十三届全国人民代表大会财政经济委员会关于国民经济和社会发展第十四个五年规划和2035年远景目标纲要草案的审查结果报告
（2021年3月9日第十三届全国人民代表大会第四次会议主席团第二次会议通过）

十三届全国人大四次会议主席团：
　　第十三届全国人民代表大会第四次会议审查了国务院提出的《中华人民共和国国民经济和社会发展第十四个五年规划和2035年远景目标纲要（草案）》（以下简称《纲要草案》）。全国人民代表大会财政经济委员会在对《纲要草案》进行初步审查的基础上，根据各代表团和有关专门委员会的审查意见，又作了进一步审查。国务院根据审查意见对《纲要草案》作了修改。现将审查结果报告如下。
　　**一、**“十三五”规划目标任务胜利完成
　　财政经济委员会认为，“十三五”时期我国经济社会发展取得新的历史性成就。面对错综复杂的国际形势、艰巨繁重的国内改革发展稳定任务特别是新冠肺炎疫情严重冲击，以习近平同志为核心的党中央团结带领全党全国各族人民砥砺前行、开拓创新，奋发有为推进党和国家各项事业，胜利完成了“十三五”规划目标任务，交出一份人民满意、世界瞩目、可以载入史册的答卷。总体来看，经济运行总体平稳，经济结构持续优化，科技创新取得重大进展，改革开放实现重要突破，脱贫攻坚成果举世瞩目，生态环境明显改善，民生得到有力保障，社会事业全面发展。经过五年持续奋斗，我国经济实力、科技实力、综合国力和人民生活水平跃上新的大台阶，决胜全面建成小康社会取得决定性成就，中华民族伟大复兴向前迈出了新的一大步。这充分彰显了中国共产党领导和中国特色社会主义制度优势，将激励全党全国各族人民再接再厉，向实现第二个百年奋斗目标继续奋勇前进。
　　当前和今后一个时期，我国仍处于重要战略机遇期，机遇和挑战都有新的发展变化。国际环境日趋复杂，不稳定性不确定性明显增加。我国发展不平衡不充分问题仍然比较突出，重点领域关键环节改革任务仍然艰巨，创新能力亟待增强，农业基础还不稳固，城乡区域发展不够平衡，收入分配差距较大，生态环保任重道远，民生保障尚存短板，社会治理还有弱项。要增强忧患意识，保持战略定力，坚定必胜信心，集中力量办好自己的事情，善于在危机中育先机、于变局中开新局，推动经济社会高质量发展。

　　**二、**《纲要草案》总体可行
　　财政经济委员会认为，国务院提出的《纲要草案》以习近平新时代中国特色社会主义思想为指导，深入贯彻落实习近平总书记在党的十九届五中全会上的重要讲话精神，符合《[中共中央关于制定国民经济和社会发展第十四个五年规划和二〇三五年远景目标的建议](https://www.pkulaw.com/chl/62a4170c41f8f103bdfb.html)》提出的目标和要求，体现了立足新发展阶段、贯彻新发展理念、构建新发展格局、推动高质量发展的要求，符合经济社会发展实际，安排总体可行。建议第十三届全国人民代表大会第四次会议批准国务院提出的《中华人民共和国国民经济和社会发展第十四个五年规划和2035年远景目标纲要（草案）》。

　　**三、**做好“十四五”时期经济社会发展工作的建议
　　“十四五”时期是我国全面建成小康社会、实现第一个百年奋斗目标之后，乘势而上开启全面建设社会主义现代化国家新征程、向第二个百年奋斗目标进军的第一个五年，做好“十四五”时期经济社会发展工作意义重大。要高举中国特色社会主义伟大旗帜，深入贯彻党的十九大和十九届二中、三中、四中、五中全会精神，坚持以马克思列宁主义、毛泽东思想、邓小平理论、“三个代表”重要思想、科学发展观、习近平新时代中国特色社会主义思想为指导，全面贯彻党的基本理论、基本路线、基本方略，统筹推进经济建设、政治建设、文化建设、社会建设、生态文明建设的总体布局，协调推进全面建设社会主义现代化国家、全面深化改革、全面依法治国、全面从严治党的战略布局，增强“四个意识”、坚定“四个自信”、做到“两个维护”，坚定不移贯彻创新、协调、绿色、开放、共享的新发展理念，坚持稳中求进工作总基调，以推动高质量发展为主题，以深化供给侧结构性改革为主线，以改革创新为根本动力，以满足人民日益增长的美好生活需要为根本目的，统筹发展和安全，加快建设现代化经济体系，加快构建以国内大循环为主体、国内国际双循环相互促进的新发展格局，推进国家治理体系和治理能力现代化，实现经济行稳致远、社会安定和谐，为全面建设社会主义现代化国家开好局起好步。为此，财政经济委员会提出以下建议：
　　（一）坚持创新驱动发展战略，发展现代产业体系。强化国家战略科技力量，把科技自立自强作为国家发展的战略支撑。健全新型举国体制，打好关键核心技术攻坚战。加强基础研究，注重原始创新。完善科技创新体制机制，加强知识产权保护，激发人才创新活力，调动全社会特别是企业创新积极性，加大研发投入，完善稳定支持机制和创新服务体系，提高科技成果转化率和资金使用效益。推进产业基础高级化、产业链现代化，提高经济质量效益和核心竞争力。保持制造业比重基本稳定，发展壮大战略性新兴产业，促进先进制造业和现代服务业深度融合。加快数字化和智能化发展，推动产业数字化转型。
　　（二）形成强大国内市场，加快构建新发展格局。坚持扩大内需这个战略基点，深化供给侧结构性改革，加强需求侧管理，培育完整内需体系。优化供给结构，改善供给质量，提升供给体系的韧性和对国内需求的适配性。打破行业垄断和地方保护，贯通生产、分配、流通、消费各环节。依托国内大循环吸引全球资源要素，充分利用国内国际两个市场两种资源，强化国内大循环主导作用，以国际循环提升国内大循环效率和水平，实现国内国际双循环相互促进。
　　（三）促进城乡区域协调发展，提升新型城镇化质量。全面推进乡村振兴，加快农业农村现代化。强化以工补农、以城带乡，推动形成工农互促、城乡互补、协调发展、共同繁荣的新型工农城乡关系。巩固提升脱贫攻坚成果，健全防止返贫监测和帮扶机制。深入实施区域重大战略、区域协调发展战略、主体功能区战略，健全区域战略统筹、市场一体化发展、区域合作互助、区际利益补偿等机制，更好地促进发达地区和欠发达地区、东中西部和东北地区共同发展。坚持陆海统筹，发展海洋经济。扎实推进以人为核心的新型城镇化，健全农业转移人口市民化机制，完善城镇化空间布局，开展城市更新行动，全面提升城市品质。
　　（四）推动绿色低碳发展，加强生态文明建设。坚持山水林田湖草系统治理，推进生态系统保护和修复。深入打好污染防治攻坚战，强化多污染物协同控制和区域协同治理，完善市场化、多元化生态补偿，持续改善环境质量。积极应对气候变化，抓紧制定2030年前碳排放达峰行动方案，完善能源消费总量和强度双控制度，加快发展方式绿色转型。积极参与和引领应对气候变化等生态环保国际合作。健全现代生态环境治理体系，建立地上地下、陆海统筹的生态环境治理制度。
　　（五）全面深化改革，推进高水平对外开放。发挥全面深化改革在构建新发展格局中的关键作用。坚持和完善社会主义基本经济制度，建设高标准市场体系，强化竞争政策基础地位。加快转变政府职能，构建市场化、法治化、国际化营商环境。深化国资国企改革，加快国有经济布局优化和结构调整。优化民营经济发展环境，依法平等保护民营企业产权和企业家权益，完善促进中小微企业和个体工商户发展的法律环境和政策体系。加快完善现代财税体制，健全政府债务管理，优化税制结构，落实税收法定原则。推动建立现代金融体系，构建金融有效支持实体经济的体制机制，完善资本市场基础制度，提高直接融资比重，坚持金融创新必须在审慎监管前提下有序进行。全面提高对外开放水平，推动贸易和投资自由化便利化，稳妥推进金融领域开放，推动共建“一带一路”高质量发展。高举构建人类命运共同体旗帜，积极参与全球治理体系改革和建设。
　　（六）增进民生福祉，扎实推动共同富裕。提高基本公共服务均等化水平，推动城乡、区域基本公共服务有效衔接。强化就业优先政策，扩大就业容量，提高就业质量，缓解结构性就业矛盾，保障劳动者权益。保持居民收入增长和经济增长基本同步，提高劳动报酬在初次分配中的比重，持续提高低收入群体收入，扩大中等收入群体，缩小收入差距。建设高质量教育体系，办好让人民满意的教育。繁荣发展文化事业和文化产业。健全覆盖全民、统筹城乡、公平统一、可持续的多层次社会保障体系，加快实现基本养老保险全国统筹，推动基本医疗、失业、工伤保险省级统筹，稳步建立长期护理保险制度。积极应对人口老龄化，优化生育政策，以“一老一小”为重点完善人口服务体系，健全普惠性学前教育和托育服务保障机制。构建强大公共卫生体系，完善基层医疗卫生保障，提高应对突发公共卫生事件和防治重大疾病能力，推动中医药传承创新，全面推进健康中国建设。
　　（七）统筹发展和安全，实现安全发展。坚持总体国家安全观，加强国家安全体系和能力建设。确保国家经济安全，增强产业体系抗冲击能力。确保粮食安全，提高良种自主可控能力。保障能源资源安全，加强战略性矿产资源规划管控。维护财政金融安全，压实监管责任，守住不发生系统性风险底线。确保生态安全，严密防控环境风险。维护新型领域安全，全面加强网络安全保障体系和能力建设。保障人民生命安全，强化安全生产和食品药品安全责任制，完善国家应急管理体系，提升灾害防治能力。维护社会稳定和安全，加强和创新社会治理，提升国家治理效能。
　　以上报告，请审议。

第十三届全国人民代表大会财政经济委员会
　　2021年3月9日

© 北大法宝：（[www.pkulaw.com](https://www.pkulaw.com/)) 专业提供法律信息、法学知识和法律软件领域各类解决方案。北大法宝为您提供丰富的参考资料，正式引用法规条文时请与标准文本核对。
欢迎查看所有[产品和服务](https://www.pkulaw.com/helps/2.html)。法宝快讯：[如何快速找到您需要的检索结果？](https://www.pkulaw.com/helps/70.html)[法宝](https://www.pkulaw.com/helps/69.html)V6有何新特色？

扫描二维码阅读原文

**原文链接：**<https://www.pkulaw.com/chl/19f6fa1be476011fbdfb.html>
